# Germplasm characterization and SDS-PAGE analysis of caper (*Capparis spinosa* L.) from different provenances

**DOI:** 10.1186/s12870-023-04620-1

**Published:** 2023-12-11

**Authors:** Min Wang, Xiaolu Yuan, Liping Xu

**Affiliations:** College of Biological Sciences and Technology, Institute of Resources and Ecology, Yili Normal University, Yining, Xinjiang China Jiefang West Road, Yining, Ili Kazakh Autonomous Prefecture, 835000

**Keywords:** *Capparis spinosa* L., Morphological characteristics, Correlation analysis, PCA analysis, SDS-PAGE

## Abstract

**Background:**

*Capparis spinosa* L. is a typical desert plant that is resistant to high temperatures and drought, and at the same time is rich in medicinal and food values. The objective of this study is to explore the variations in nutrient composition, morphological characteristics, and SDS-PAGE patterns of caper seeds from different provenances, aiming to provide insights for the selection of superior seed provenances.

**Results:**

In this experiment, there were significant differences in the morphological characteristics and major nutritional components of caper seeds from different provenances. Seeds from the YKL (Karayagaqi Township, Yining County) and YKG (G218, KashiTown, Yining County) regions were larger in size compared to seeds from other regions. Among the four measured nutritional components, crude fat had the highest content, especially in the YKL and YKG region. The results of correlation analysis showed that crude fat was negatively correlated with soluble sugar and soluble protein but significantly positively correlated with starch content. As longitude increased from east to west, the morphological characteristics gradually increased. Based on the principal component analysis of all the parameters of the seeds, the eight provenances could be classified into three groups. HM (Hami), TGS (S202, Gaochang District, Turpan), HYW (Wubao Town, Yizhou District, Hami), TQQ (Qiquanhu Town, Turpan), and TLF (Turpan) were a group with higher soluble protein, soluble sugar, and water content. YKL and YKG were in one group, which had larger seed grains with high crude fat and starch content. AKS (Aksu) was in a separate group. The protein fractions from seeds of eight regions were extracted using Osborne fractionation method, it was found that glutelin content was the highest, while albumin content was the lowest. After these proteins were analyzed by SDS-PAGE, the electrophoretic patterns showed that the protein molecular weights were relatively small, and there were differences in protein bands among different provenances.

**Conclusion:**

According to the PCA results, the eight seed provenances could be divided into three groups. There were both geographically distant ones clustered into one group, and those close to each other were also divided into one group. There were differences in seed morphology, nutrient content and SDS-PAGE profiles among the different seed sources. This difference might be caused by a combination of geographic and climatic factors. In addition, YKL and YKG were roughly selected as good seed provenances, which provided a theoretical basis for the development of *C. spinosa* L. germplasm resources.

**Supplementary Information:**

The online version contains supplementary material available at 10.1186/s12870-023-04620-1.

## Introduction

*Capparis spinosa* L., commonly known as the caper bush, is a perennial plant belonging to the Capparaceae family [1]. It thrives in dry regions such as tropical and subtropical areas, with a particularly wide distribution in the Mediterranean region [2]. In China, it is found in regions like Tibet, Gansu, and Xinjiang [3, 4]. This plant is known for its ability to withstand arid and high-temperature conditions, tolerating temperatures exceeding 40 °C. Its well-developed root system enables efficient water absorption [5], resulting in higher water use efficiency compared to other desert plants [6]. The creeping branches of *C. spinosa* grow close to the ground and can form dense shrubs with a diameter exceeding 2 meters [7]. This characteristic increases the surface area in contact with the ground, enhancing surface roughness and effectively reducing wind speed, preventing wind erosion and sand movement [8–11]. Consequently, this plant is beneficial for combating soil erosion in arid and semi-arid regions [12, 13].

*C. spinosa* has been utilized for various purposes throughout history. It can be used as food, food additives [11], and in the production of cosmetics [14]. Additionally, it is valued as a traditional medicinal herb due to its anti-inflammatory, analgesic, antibacterial, antioxidant, and hypoglycemic properties [15–18]. The chemical composition of *C. spinosa* is also a subject of research interest, with components such as flavonoids (quercetin, rutin, etc.) [19, 20], alkaloids (choline, sophoramine) [21, 22], fatty acids [23–25], and volatile oils [26–28]. These characteristics highlight the ecological and significant commercial value of *C. spinosa*.

Awatef et al. [29] conducted a study on the morphological variations of *C. spinosa* seeds among 17 different seed provenances. They observed significant morphological differences among the seed provenances and performed cluster analysis based on morphological parameters, categorizing the provenances into two groups. Furthermore, researchers investigating the impact of different geographical locations on the bioactivity and related functions of *C. spinosa* components found that among three seed provenances from Italy, Morocco, and Turkey, Moroccan *C. spinosa* exhibited the highest content of polyphenolic compounds, followed by Italy and Turkey [30]. There is a considerable correlation between the genetic differentiation of *C. spinosa* and its geographical distribution. Wang et al. [31] explored the effect of geographic separation on the differentiation pattern of caper species in arid regions from a molecular perspective. About 300 individuals were sampled from 25 caper populations, and 14 haplotypes are identified. And the AMOVA results show that significant genetic differentiation has occurred between populations, indicating a considerable correlation between genetic differences and geographic distribution, and the isolation of complex mountain and desert geography may limit gene exchange between segregating populations, resulting in high divergence between populations.

SDS-PAGE is a commonly used electrophoretic technique using polyacrylamide gel as a supporting medium, which is widely employed for protein molecule separation. Li et al. [32] utilized SDS-PAGE to separate the glycoprotein components in ginkgo seeds and 11 glycoproteins were isolated from ginkgo seeds. Some researchers have employed SDS-PAGE analysis to demonstrate that Turkish coriander varieties lack population structure and genetic bottlenecks. Based on these findings, it is possible to refine the sampling strategy to initiate an effective coriander breeding program in Turkey [33]. Zhang et al. [34]. utilized a comprehensive array of techniques, including SDS-PAGE, RP-HPLC, MALDI-TOF, and peptide sequencing, to accurately isolate and identify various homologous avenin-like proteins.

Currently, *C. spinosa* remains in a wild state and has not yet been extensively cultivated in China. Moreover, this plant exhibits an extremely low germination rate under natural conditions, which limits its genetic resources. However, it possesses abundant ecological, medicinal, and culinary value. Therefore, the purpose of this study is to select superior provenances by comparing the morphological characteristics and nutritional differences of caper seeds from different origins. This selection is conducive to identifying and exploring optimal seed production areas for caper. Furthermore, this study employs the Osborne fractionation method for the first time to extract proteins from caper seeds and subsequently conducts SDS-PAGE analysis. This analysis serves as a reference for future research on the protein components of caper seeds.

## Results

### ANVOA analysis of morphological characteristics of caper seeds

To investigate the morphological differences in caper seeds from different provenances, a one-way analysis of variance (ANOVA) was conducted on the data, as shown in Table 1. The results revealed significant variations in various morphological parameters among the seed provenances. Thousand-seed weight ranged from 1.4474 g (TLF) to 7.1490 g (YKG), showing a 4.94-fold difference in size. Seed moisture content varied from 5.37% (AKS) to 10.90% (HM), exhibiting a 2.03-fold difference. Seed length ranged from 0.23 cm (HYW) to 0.32 cm (YKL), showing a 1.39-fold difference. Seed width varied from 0.16 cm (TLF) to 0.25 cm (YKL), with a 1.56-fold difference. Seed thickness ranged from 0.01 cm (AKS) to 0.19 cm (YKL), exhibiting a 19-fold difference. Particle size varied from 0.16 cm (TLF) to 0.25 cm (YKL), with a 1.56-fold difference. Sphericity ranged from 0.69 (TLF) to 0.78 (AKS), with a 1.13-fold difference. Surface area varied from 0.09 cm^2^ (TQQ) to 0.20 cm^2^ (YKL), exhibiting a 2.22-fold difference. Volume ranged from 0.002 cm^3^ (HYW, HM, TQQ) to 0.01 cm^3^ (YKL, YKG), showing a fivefold difference. Seed viability varied from 0.20% (HYW) to 0.87% (YKL), exhibiting a 4.35-fold difference. Fruit length ranged from 1.88 cm (HM) to 3.34 cm (YKL), with a 1.78-fold difference. Fruit width varied from 1.07 cm (HM) to 1.48 cm (YKL), showing a 1.48-fold difference. Seed color was predominantly blackish-brown, yellowish-brown, or brown, except for YKG and YKL, which had a grayish-brown color. Notably, the most significant differences were observed in thousand-seed weight and seed thickness. The measurement results indicated that the seeds from the YKL and YKG provenances were the largest in terms of morphology.

**Table 1:** Morphological characteristics of different provenances

Provenance	Rate of water content (%)	Single grain weight(g)	Thousand seed weight (g)	Seed length (cm)	Seed width (cm)	Seed thickness(cm)	Diameter(cm)
AKS	5.37 ± 0.006Bb	0.0042 ± 0.001Bb	1.6785 ± 0.058Ee	0.27 ± 0.027Bb	0.22 ± 0.022Bb	0.01 ± 0.012Bb	0.21 ± 0.010Bb
HYW	6.38 ± 0.001Bb	0.003 ± 0.001BCbc	2.2847 ± 0.089Dd	0.23 ± 0.023Cc	0.18 ± 0.021Cc	0.14 ± 0.012Cc	0.18 ± 0.011Cc
HM	10.90 ± 0.016Aa	0.0018 ± 0.001Cc	2.4297 ± 0.216CDcd	0.25 ± 0.022BCbc	0.17 ± 0.020Cc	0.13 ± 0.021CDEcde	0.18 ± 0.010CDcd
TQQ	5.67 ± 0.001Bb	0.0022 ± 0.001Cc	2.7141 ± 0.077Cc	0.24 ± 0.022Cc	0.17 ± 0.024Cc	0.13 ± 0.012DEde	0.17 ± 0.011CDcd
TLF	9.36 ± 0.002Aa	0.0024 ± 0.001Cc	1.4474 ± 0.044Ee	0.24 ± 0.032Cc	0.16 ± 0.037Cc	0.12 ± 0.017Ee	0.16 ± 0.021Dd
TGS	6.37 ± 0.04Bb	0.0024 ± 0.001Cc	2.4304 ± 0.225CDcd	0.25 ± 0.025C	0.17 ± 0.033Cc	0.14 ± 0.013CDcd	0.18 ± 0.013Cc
YKL	6.47 ± 0.006Bb	0.0076 ± 0.003Aa	5.9384 ± 0.259Bb	0.32 ± 0.022Aa	0.25 ± 0.028Aa	0.19 ± 0.014Aa	0.25 ± 0.013Aa
YKG	5.98 ± 0.015Bc	0.0074 ± 0.001Aa	7.1490 ± 0.133Aa	0.31 ± 0.026Aa	0.24 ± 0.030ABab	0.18 ± 0.013Aa	0.24 ± 0.016Aa
Average	7.06 ± 0.018	0.0039±0.003	3.26 ± 1.959	0.27 ± 0.035	0.20 ± 0.037	0.13 ± 0.055	0.20 ± 0.033

Provenance	Sphericity	Surface area (cm^2^)	Volume (cm^3^)	Seed vigor (%)	Fruit length (cm)	Fruit width (cm)	Seed colour
AKS	0.78 ± 0.06Aa	0.14 ± 0.014Bb	0.004 ± 0.0006Bb	0.38 ± 0.02Cc	2.74 ± 0.440Bb	1.22 ± 0.138BCbc	Dark brown,brown
HYW	0.76 ± 0.047ABab	0.10 ± 0.013Cc	0.002 ± 0.0004Cc	0.20 ± 0.055Dd	2.30 ± 0.348Cc	1.27 ± 0.215Bb	Dark brown,brown
HM	0.71 ± 0.079BCbc	0.10 ± 0.011Cc	0.002 ± 0.0004Cc	0.27 ± 0.031CDcd	1.88 ± 0.271Dd	1.07 ± 0.142Dd	Dark brown, yellowish-brown
TQQ	0.71 ± 0.059BCbc	0.09 ± 0.012Cc	0.002 ± 0.0004Cc	0.38 ± 0.045Cc	2.07 ± 0.280CDcd	1.12 ± 0.166CDcd	Dark brown, yellowish-brown
TLF	0.69 ± 0.071Cc	0.09 ± 0.022Cc	0.002 ± 0.0007Cc	0.34 ± 0.085CDcd	2.21 ± 0.317CDcd	1.16 ± 0.096BCDbcd	Dark brown, yellowish-brown
TGS	0.73 ± 0.075ABCabc	0.10 ± 0.014Cc	0.002 ± 0.0006Cc	0.59 ± 0.172Bb	2.26 ± 0.423CDcd	1.12 ± 0.141CDcd	Dark brown, yellowish-brown
YKL	0.76 ± 0.053ABab	0.20 ± 0.021Aa	0.01 ± 0.001Aa	0.87 ± 0.064Aa	3.34 ± 0.440Aa	1.48 ± 0.166Aa	Taupe
YKG	0.77 ± 0.053Aa	0.18 ± 0.024Aa	0.01 ± 0.001Aa	0.87 ± 0.031Aa	2.76 ± 0.590Bb	1.28 ± 0.233BCbc	Taupe
Average	0.74 ± 0.035	0.12 ± 0.043	0.003 ± 0.002	0.49 ± 0.263	2.44 ± 0.472	1.22 ± 0.133	None

All data in the table are average ± SD. Uppercase letter indicates significant differences at the* p* < *0.01* level. Lowercase letters indicates significant differences at the* p* < *0.05* level

### Analysis of nutrient content of caper seeds

This study conducted an analysis of the nutritional composition differences in caper seeds from different provenances. The significance of the data variations is presented in detail in Table 2. The YKG exhibited the highest crude fat content at 34.42%, while the lowest content was found in the TLF at 13.09%, showing a 2.63-fold difference. The highest soluble sugar content was observed in the AKS at 11.31%, whereas the lowest content was found in the TLF at 5.33%, resulting in a 2.12-fold difference. The TLF showed the highest soluble protein content at 1.83 g/100 g, while the lowest content was found in the TGS at 0.37 g/100 g, showing a 4.95-fold difference. The highest starch content was observed in the YKL at 4.36%, whereas the lowest content was found in the TLF at 1.43%, resulting in a 3.05-fold difference. It is evident that crude fat is the dominant nutritional component in caper seeds, followed by soluble sugars, starch, and protein. Among these four nutritional components, crude fat content exhibited the most significant differences, while the differences in the other three nutritional components were not statistically significant. Furthermore, crude fat is the nutrient component with the highest content. Moreover, the highest nutrient content of caper seeds is crude fat, and the provenance with the highest crude fat content is the YKG.

**Table 2:** Nutrient component characteristics of caper seeds from different provenances

Provenance	Crude fat(%)	Soluble sugar(%)	Starch(%)	Soluble protein(g/100 g)
AKS	24.75 ± 0.006Cc	11.31 ± 0.006Aa	4.29 ± 0.006Aa	1.53 ± 0.001Aa
HYW	22.09 ± 0.013CDcd	10.71 ± 0.008ABab	2.32 ± 0.008ABab	1.29 ± 0.004ABab
HM	16.16 ± 0.033DEde	10.21 ± 0.008ABab	1.80 ± 0.0070Bb	1.54 ± 0.0004Aa
TQQ	25.58 ± 0.016BCbc	7.38 ± 0.033ABab	2.37 ± 0.04ABab	1.70 ± 0.001Aa
TLF	13.09 ± 0.042Ee	5.33 ± 0.047Bb	1.43 ± 0.017Bb	1.83 ± 0.001Aa
TGS	25.13 ± 0.013Cc	7.21 ± 0.009ABa	2.57 ± 0.007ABab	0.37 ± 0.001Cc
YKL	31.34 ± 0.011ABab	5.46 ± 0.011Bb	4.36 ± 0.003Aa	0.83 ± 0.004BCbc
YKG	34.42 ± 0.034Aa	6.07 ± 0.015ABab	4.22 ± 0.005Aa	0.80 ± 0.002BCbc
Average	24.07 ± 0.066	7.96 ± 0.023	2.92 ± 0.012	1.24 ± 0.005

All data in the table are average ± SD. Uppercase letter indicates significant differences at the* p* < *0.01* level. Lowercase letters indicates significant differences at the *p* < *0.05* level

### Pearson correlation analysis of nutritional components and morphological characteristics in caper seeds

**Fig. 1:**
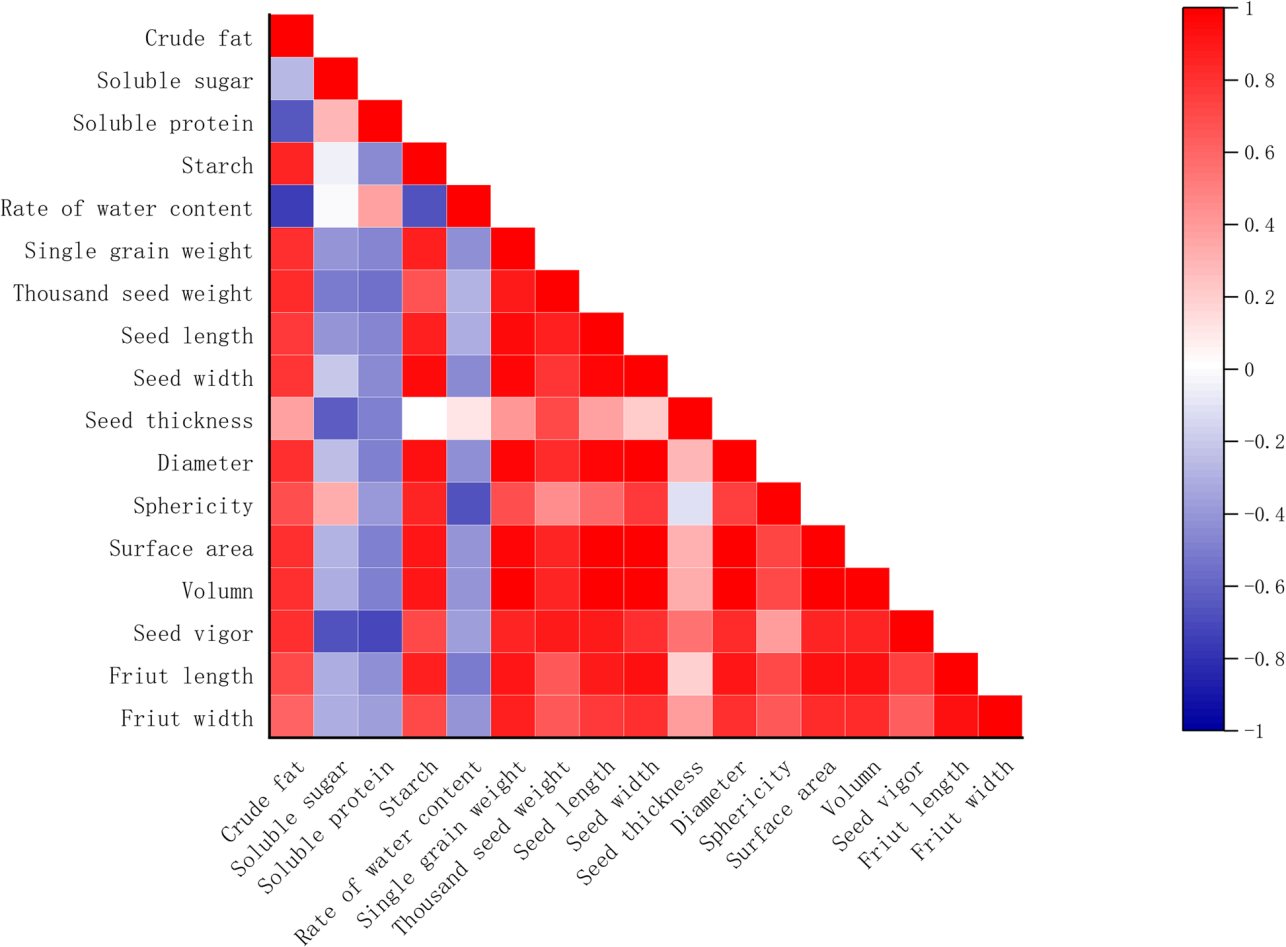
Correlation heat map of nutrients and morphological characteristics of caper seeds

The correlation analysis was performed on the major nutritional components and morphological characteristics of caper seeds, and the data results were plotted using Origin 2021 software (Fig. 1). Thousand-seed weight and individual seed weight showed positive correlations with seed length, seed width, seed thickness, particle size, sphericity, surface area, volume, fruit length, and fruit width. They also exhibited significant correlations with seed width and particle size (*p* < *0.05*), and extremely significant correlations with individual seed weight, seed width, and surface area (*p* < *0.01*). Seed length showed significant correlations with fruit width (*p* < *0.05)* and extremely significant correlations with seed width, particle size, surface area, volume, and fruit length (*p* < *0.01*). Seed width exhibited significant correlations with sphericity and fruit length–width ratio (*p* < *0.05*), and extremely significant correlations with particle size, surface area, and volume (*p* < *0.01*). Seed thickness showed negative correlations with sphericity and surface area, and positive correlations with particle size, volume, and fruit length–width ratio, but these correlations were not statistically significant. Particle size showed significant correlations with sphericity and fruit width (*p* < *0.05*), and extremely significant correlations with surface area, volume, and fruit length (*p* < *0.01*). Sphericity showed significant correlations with surface area and volume (*p* < *0.05*), but no significant correlation with fruit width. Surface area exhibited a significant correlation with fruit width (*p* < *0.05*), and extremely significant correlations with volume and fruit length (*p* < *0.01*). Volume showed a significant positive correlation with fruit width and an extremely significant positive correlation with fruit length (*p* < *0.01*). Fruit length showed an extremely significant positive correlation with fruit width (*p* < *0.01*).

The moisture content showed an extremely significant negative correlation with thousand-seed weight (*p* < *0.01*). It exhibited no significant correlation with seed thickness but showed negative correlations with other morphological parameters, although these correlations were not statistically significant. Seed viability showed positive correlations with all 11 morphological parameters. It exhibited extremely significant positive correlations with individual seed weight, thousand-seed weight, seed length, volume, and surface area (*p* < *0.01*), and significant positive correlations with seed width and particle size (*p* < *0.05*). It showed a negative correlation with moisture content, but the correlation was not statistically significant.

In terms of the four nutritional components, crude fat showed negative correlations with soluble sugars and soluble proteins, but these correlations were not statistically significant. It exhibited an extremely significant negative correlation with starch content (*p* < *0.01*). Soluble sugars showed a weak positive correlation with soluble proteins and a negative correlation with starch content, but these correlations were not statistically significant. Soluble proteins also showed a negative correlation with starch content, but it was not statistically significant.

The correlation analysis between nutritional components and morphological characteristics (Fig. 1) reveals the following relationships: Crude fat exhibited positive correlations with morphological parameters and showed significant correlations (*p* < *0.05*) with individual seed weight, seed length, seed width, particle size, sphericity, surface area, and fruit length. Soluble sugars showed a positive correlation with sphericity but negative correlations with other morphological parameters, although these correlations were not statistically significant. Soluble proteins exhibited negative correlations with morphological parameters, but these correlations were not statistically significant. Starch showed positive correlations with morphological parameters, except for seed thickness and fruit width, where the correlations were not significant. These correlations were extremely significant (*p* < *0.01*). Seed moisture content showed a significant negative correlation with crude fat (*p* < *0.05*) and no significant correlation with soluble sugars or starch. It exhibited a nonsignificant correlation with soluble proteins. Seed viability showed a negative correlation with soluble sugars and proteins, but these correlations were not statistically significant. It exhibited significant correlations (*p* < *0.05*) with crude fat and starch.

### Pearson correlation analysis of nutritional composition and morphological characteristics of caper seeds with geographical factors

**Fig. 2:**
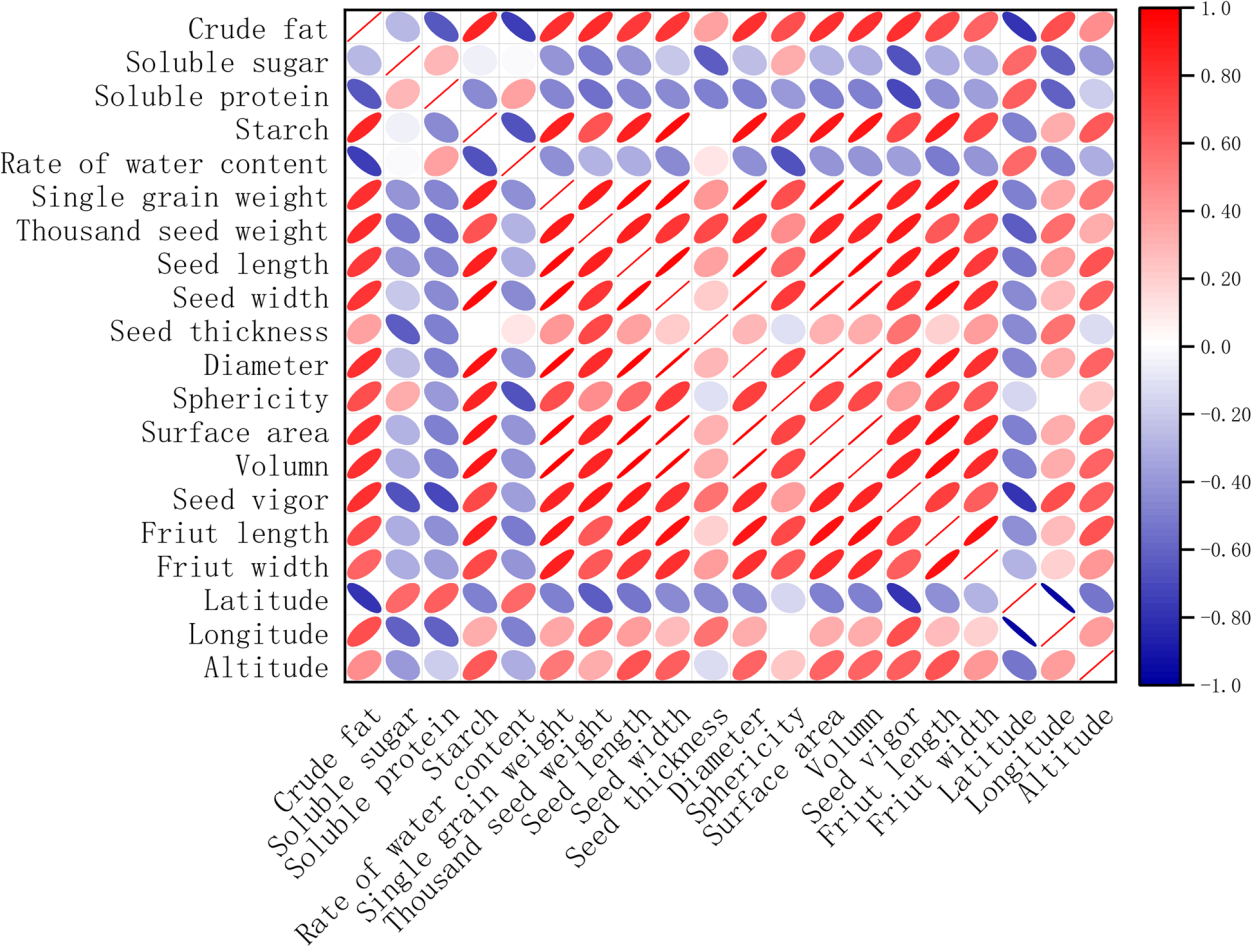
Correlation heat map of nutritional composition and morphological characteristics of caper seeds with geographical factors

The correlation analysis was conducted to examine the relationship between the nutritional composition, morphological characteristics, and geographical factors of caper seeds (Fig. 2). The correlation between seed composition, morphology, and geographical location was found to be intricate. As longitude gradually shifted from east to west, the morphological parameters exhibited a positive increase, indicating a larger seed size and higher vitality. However, the content of soluble sugars, soluble proteins, and moisture decreased accordingly. On the other hand, as latitude moved from south to north, the morphological characteristics of the seeds showed a negative correlation. This resulted in a gradual decrease in seed size and a decline in vitality. The content of crude fat and starch also decreased, while other components showed a positive correlation. Furthermore, with increasing altitude, there was a positive correlation observed between seed morphology indicators and seed vitality. Except for soluble sugars and soluble proteins, which showed a positive correlation, all other seed composition parameters exhibited a negative correlation. Notably, there was a significant negative correlation between crude fat content and latitude.

### Principal component analysis of morphological characteristics and nutritional composition of caper seeds from different districts

**Fig. 3:**
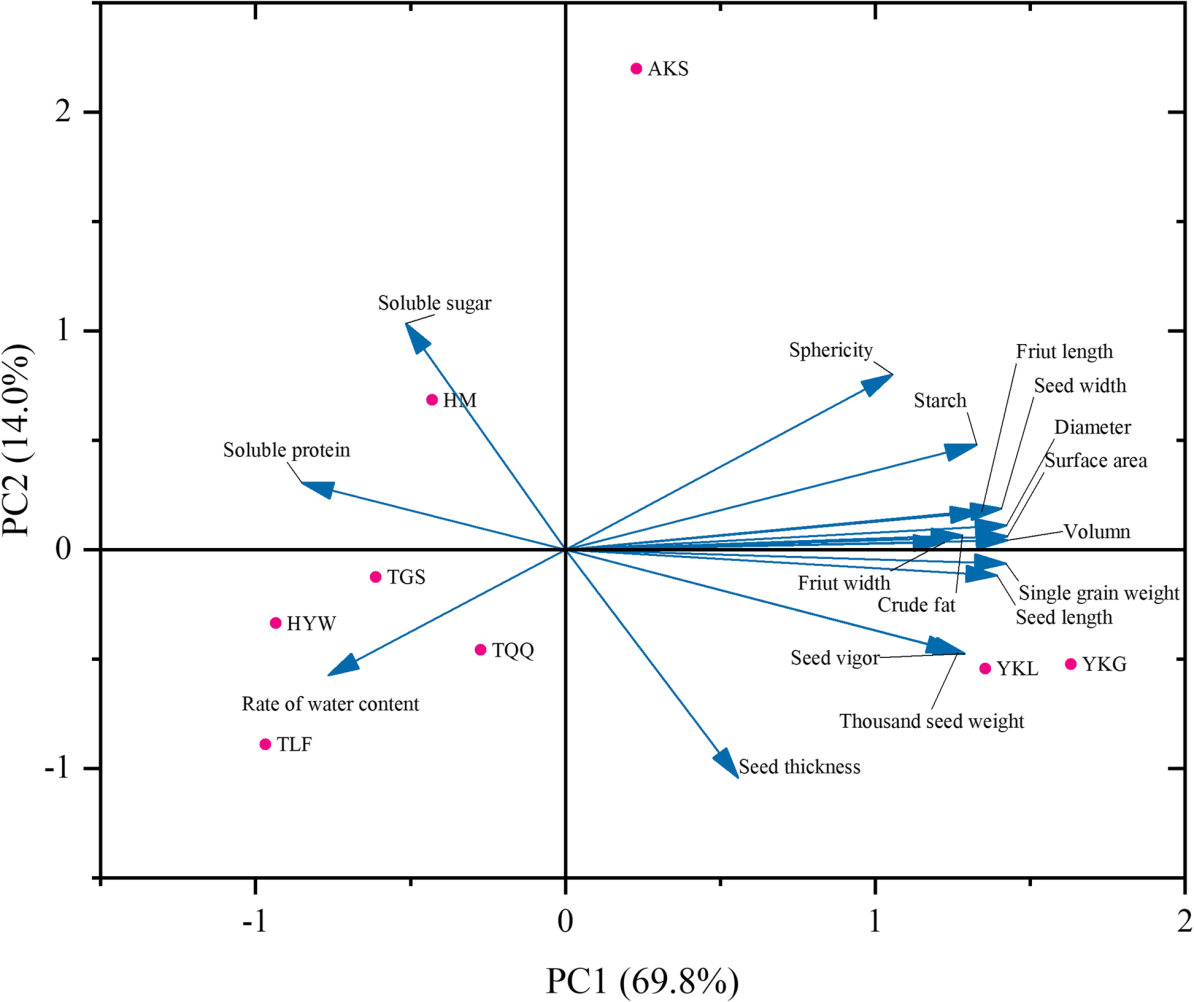
Principal component analysis (PCA) of caper seeds of morphological characteristics and nutritional composition

Principal component analysis (PCA) biplot, with the morphological indices and major nutritional components of caper seeds from eight different regions have been shown in Fig. 3. The figure visually illustrates which indicators dominate the seeds from different regions and three well-differentiated groups could be observed. In the left quadrant five provenances (HM, TGS, HYW, TQQ, TLF) were grouped. These provenances had higher levels of soluble protein, soluble sugar, and moisture content. Next, in the lower right quadrant, the YKL and YKG provenances were associated with larger seed size, crude fat and starch content. Finally, the third group consisted of the AKS provenance, which was located far from the other provenances in the upper right quadrant, indicating significant differences in seed morphology and composition compared to the other provenances.

### Protein content of caper seeds from different districts

**Fig. 4:**
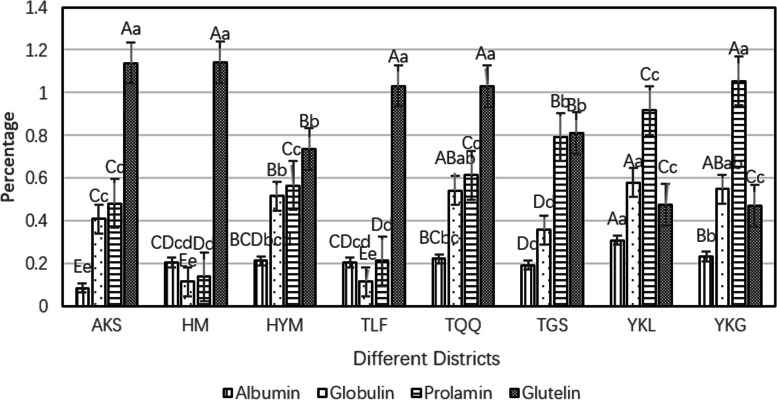
Contents of four proteins in caper seeds from different districts Note: Upper case letters indicate significant differences at the 0.01 level; lower case letters indicate significant differences at the 0.05 level

The protein content of four protein fractions extracted using the Osborne classification method varied significantly among the eight germplasm provenances (Fig. 4). Among the germplasm provenances, YKL had the highest content of albumin at 0.23%, while AKS had the lowest content at 0.08%. YKL also had the highest content of globulin at 0.58%, while HM had the lowest at 0.11%. YKG had the highest content of glutelin at 1.05%, while HM had the lowest at 0.14%. HM had the highest content of alcohol soluble protein at 1.14%, while YKG had the lowest at 0.47%. Among these germplasm provenances, the highest protein fraction content was observed for glutelin in AKS, HM, HYW, TLF, TQQ, and TGS, while the highest content of albumin was found in YKL and YKG. The content of protein fractions also showed variability in different provenances.

### Overall mean protein content of the four protein fractions

**Fig. 5:**
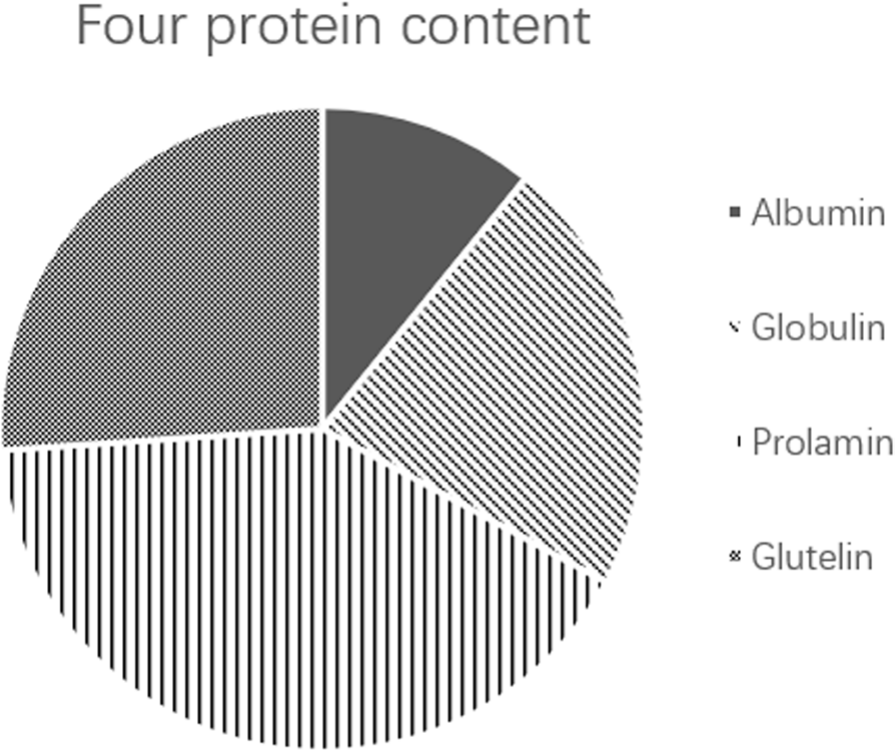
The total amount of the four proteins

The overall mean protein content of the four protein fractions in these eight germplasm provenances is illustrated in Fig. 5. Among the four protein fractions, glutelin had the highest average content at 0.85%, while albumin had the lowest average content at 0.21%. The content of alcohol-soluble protein was the next highest, with an average of 0.60%, followed by globulin with an average content of 0.40%.

### SDS-PAGE analysis of the four protein fractions from different districts

**Fig. 6:**
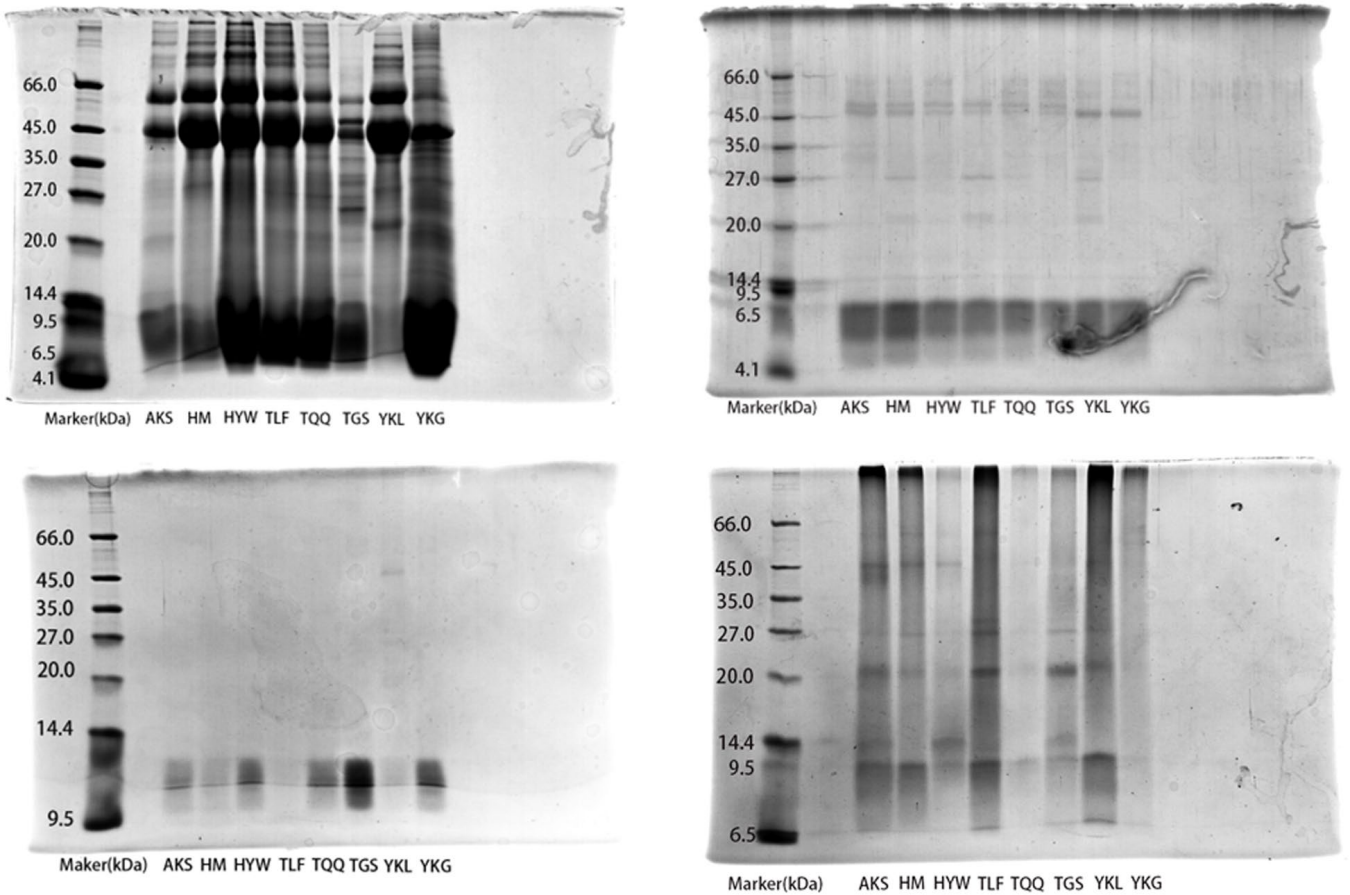
SDS-PAGE mapping of seed proteins from different provenances (**a**: albumin, **b**: globulin, **c**: alcohol soluble protein, **d**: glutelin)

Electrophoresis analysis was conducted on the four protein fractions extracted using the Osborne classification method from caper seeds. The differences in protein profiles among different germplasm provenances were observed in terms of band thickness and color intensity, indicating variations not only in the types of proteins expressed but also in their abundance. The results are depicted in Fig. 6. Among the four protein profiles, albumin exhibited the highest number of bands (Fig. 6 a), mainly concentrated around 66.0, 45.0, 35.0, 27.0, and 20.0 kDa. Among these bands, AKS, HM, HYW, TLF, and TQQ shared similar distributions in the high molecular weight protein region, but showed differences in the low molecular weight protein region. The banding patterns and quantity of albumin were almost identical between TLF and TQQ. In the region above 66.0 kDa, TGS had fewer and lighter-colored bands, while YKL and YKG had fewer bands compared to other germplasm provenances. Among these eight germplasm provenances, two identical bands were observed at 66.0 kDa and 45.0 kDa. However, TGS and YKG exhibited thinner bands and lighter colors at 66.0 kDa. Compared to other germplasm provenances, TGS displayed an additional specific band at 45.0 kDa.

The number of bands in the globulin profile was noticeably fewer compared to the albumin profile (Fig. 6b). There were three common bands among the eight germplasm provenances, located at 45.0, 14.4, and 6.5 kDa. Among these bands, the ones at 45.0 kDa exhibited darker colors and wider widths in YKL and YKG compared to other germplasm provenances. The bands at 14.4 kDa were light in color and narrow in width for all germplasm provenances. In the region just above 45.0 kDa, there was a light-colored band present in AKS, HM, HYW, TLF, TQQ, and TGS. At 27.0 and 20.0 kDa, HM, TLF, and YKL exhibited two identical bands.

In the electrophoretic profile of alcohol-soluble proteins (Fig. 6c), it can be visually observed that the number of bands was the fewest. Except for YKL, the other provenances had only one band. There was one common band among all eight districts, located between 14.4 and 9.5 kDa. YKL had a specific band at 45.0 kDa.

In the electrophoretic profile of glutelin (Fig. 6d), the bands were primarily distributed at 27.0, 20.0, and 9.5 kDa. The only common band among all provenances was located at 9.5 kDa. TQQ and YKG had the fewest number of bands and the lightest colors in their profiles.

## Discussion

The same plant species exhibits genetic variations in its seeds across different distribution areas as an adaptation to diverse environments, resulting in the expression of stable genetic traits in various seed qualities [35, 36]. Variations among seed provenances also reflect the plant's geographic reproductive isolation [37]. The morphological characteristics of the caper seeds from eight provenances in this study exhibited significant differences. Seeds from the YKL and YKG regions generally exhibited the largest morphological indices. Morphological indices and seed viability mostly showed positive correlations with altitude and latitude, and a positive correlation with longitude. Based on this, it can be inferred that seed morphological differences are closely related to the geographic locations of the regions [38]. Different plant species exhibit distinct geographic patterns of seed morphology differences. For instance, *Lindera aggregata* seeds demonstrated evident regional effects, with seeds from the central region (Hunan and Jiangxi) generally being larger than those from the eastern region (Zhejiang and Anhui) (Table 3) [39]. The phenotypic characteristics of *Melia azedarach* exhibited a pronounced northeast-southwest directional difference. The length of the seeds gradually increased from south to north, while the width increased from east to west [40]. Geographic differences in the width, thickness, and weight of a hundred seeds in *Magnolia officinalis* showed a latitude gradient but were not statistically significant (Table 4) [41]. In a study of geographic variations in seed morphology and germination characteristics of licorice, it was found that seed size is significantly positively correlated with the annual rainfall at the origin, and the germination rate of licorice seeds was positively correlated with altitude. These variations are related to the adaptation of provenances to the ecological conditions of their respective origins [42].

**Table 3:** Geographic location of the provenances and morphological characteristics of *L. aggregate* seeds [39]

Number	Provenances	Longitude(E)	Latitude(N)	Hundred-grain weight(g)	Seed Length(cm)	Seed Width(cm)
1	Daoxian	111°57′	25°52′	15.007	1.022	0.706
2	Anhua	111°02′	28°38′	24.547	1.008	0.852
3	Leishan	108°07′	26°38′	19.640	1.002	0.726
4	Jianshi	109°07′	30°05′	20.487	0.998	0.826
5	Longsheng	110°02′	25°78′	14.177	0.996	0.734
6	Jianghua	111°79′	24°97′	14.213	0.982	0.730
7	Qianshan	116°53′	30°62′	19.813	0.962	0.812
8	Huoshan	116°32′	31°38′	19.573	0.954	0.854
9	Hongjiang	109°96′	27°71′	16.803	0.954	0.786
10	Lushan	115°97′	29°41′	23.683	0.948	0.846
11	Wufeng	111°06′	30°02′	23.300	0.946	0.0824
12	Rongshui	109°24′	25°07′	14.147	0.934	0.754
13	Hefeng	110°23′	30°27′	16.033	0.934	0.718
14	Tonggu	114°37′	28°53′	16.783	0.928	0.776
15	Longquan	119°13′	28°10′	17.693	0/914	0.756
16	Lichuan	108°2 l′	30°03′	15.980	0.908	0.856
17	Guangze	117°34′	27°54′	15.807	0.898	0.772
18	Sangzhi	110°16′	29°38′	16.543	0.884	0.734
19	Xishui	106°02′	28°33′	17.720	0.880	0.842

**Table 4:** Geographic location of the provenances and morphological characteristics of *M. officinalis *seeds [41]

Number	Provenances	Longitude(E)	Latitude(N)	Thousand seed weight(g)	Seed Length(mm)	Seed Width(mm)
1	Xinning, Hunan	110°51′	26°27′	80.93	6.11	5.19
2	Wuning, Jiangxi	115°05′	29°16′	65.16	5.31	4.97
3	Yifeng, Jiangxi	114°47′	28°24′	66.66	5.96	5.12
4	Gao’an, Jiangxi	115°23′	28°25′	80.90	6.11	5.12
5	Longnan, Jiangxi	114°49′	24°55′	63.15	5.82	4.88
6	Shangyou, Jiangxi	114°33′	25°48′	79.24	5.92	5.50
7	Jianggangshan,Jiangxi	114°12′	26°39′	37.45	4.57	4.59
8	Ganzhou, Jiangxi	114°55′	25°32′	72.36	5.78	4.53
9	Wuyishan, Fujian	117°38′	27°46′	45.63	5.35	4.59
10	Zhuji, Zhejiang	120°15′	29°42′	50.58	5.12	4.39
11	Tiantai, Zhejiang	120°59′	29°10′	58.61	5.56	4.43
12	Fuyang, Zhejiang	119°57′	30°03′	56.42	5.58	4.78
13	Pan’an, Zhejiang	118°05′	29°59′	60.10	5.81	5.00
14	Dongzhi, Anhui	116°59′	30°05′	49.67	5.42	4.59

Among morphological characteristics, the thousand seed weight is one of the important indicators of seed quality, reflecting the size and plumpness of seeds. A larger thousand seed weight indicates fuller seeds with richer stored nutrients, which is more favorable for seed germination [43, 44]. Clearly, the thousand seed weight of the YKL and YKG was the largest, and their germination rates were also the highest. Another important indicator of seed quality is moisture content. During seed storage, moisture content is also a significant factor affecting seed vitality [45]. Seed moisture content is a crucial factor influencing seed lifespan. Seeds with high moisture content have a shorter lifespan, are prone to mold during storage, while seeds with low moisture content have a longer lifespan and better storage tolerance. The results of this study indicated a negative correlation between seed moisture content and seed vitality. The HM had the highest moisture content, indicating lower seed vitality at 0.27%. This also suggests that seeds from the HM are not suitable for long-term storage. It is recommended to measure seed moisture content before storage to determine the storage duration and prevent mold growth during storage, leading to reduced germination rates.

The differences in the nutritional content of caper seeds from different regions were also significant. Although the seeds from the YKL and YKG had the highest morphological characteristics indices, only the crude fat content was the highest among their main stored nutrients. Therefore, it appeard that a larger thousand seed weight did not necessarily indicate higher content of a particular nutrient. Li Juan et al. [46] also explored the primary nutritional components of *Phoebe bournei* seeds from different origins. They found that starch was the main nutritional component in *Phoebe bournei* seeds, with the highest starch content in Fuchuan, Guangxi at 54.77 g/100 g and the lowest starch content in the Congjiang, Guizhou at 46.37 g/100 g. Additionally, they discovered a close correlation between starch content in *Phoebe bournei* seeds and altitude, annual precipitation, and annual average sunshine duration. In this study, the caper seeds from the YKG had the highest crude fat content at 31.34%, while seeds from the TLF had the lowest crude fat content at 13.09%. The current research only conducted a correlation analysis between seed morphological indices, major nutritional components, and geographical distribution differences, revealing that geographical location indeed contributes to genetic differences. However, it is important to note that climate factors should not be overlooked, and these differences cannot be solely attributed to geographical factors.

PCA is a multivariate statistical analysis method. This method can represent the differences in original samples by identifying two or more principal component factors. Then, based on the analysis of the contribution of these principal component factors in a large number of complex samples, it assesses the regularity and variability between samples [47]. The results of PCA analysis indicated that PC1 and PC2 contributed variances of 69.8% and 14.0%, respectively, explaining a total of 83.8% of the total variance. PC1 was correlated with most seed morphological indicators (excluding seed thickness and moisture content), crude fat, and starch. PC2 was associated with soluble sugar and seed thickness. According to the results, geographically similar provenances (YKL, YKG) clustered into one group, while geographically distant ones (HM, TGS, HYW, TQQ, TLF) clustered into another group. Considering the overall research findings, it could be concluded that the morphological characteristics and major nutritional components of caper seeds exhibit regional and random variation patterns across different provenances.

According to the results of the Coomassie Brilliant Blue method, the average protein content of caper seeds, in descending order, was: glutenin > alcohol soluble protein > globulin > albumin.

In the context of these eight production areas, it was observed that caper seeds from AKS, HM, HYW, TLF, TQQ, and TGS regions had the highest levels of glutelin, whereas seeds from YKL and YKG regions exhibited the highest content of alcohol-soluble proteins. This implied that the variations in protein composition content within the seeds might be linked to the differences in geographical locations. The soluble protein content can reflect the characteristics of plants under different geographical environmental conditions, and the quantity of soluble proteins is a result of the plant's physiological metabolism as well as a reflection of its adaptation to external environmental factors. The varying protein content among the caper seeds from these eight different regions also indirectly reflected their responses to local climate conditions such as high temperatures and drought. Due to the differences in habitat conditions, caper seeds from different provenances had developed their own relatively stable adaptive traits over prolonged exposure to distinct geographical environments. As a result, they exhibit varying degrees of drought resistance, heat tolerance, and associated mechanisms [48, 49].

In most grain protein extractions, alcohol-soluble protein and glutelin tend to have higher contents. The results of this study showed that caper seeds had high content of glutelin and alcohol-soluble protein. Alcohol-soluble protein and glutelin, also known as storage proteins, are used for seedling growth [50]. They were first discovered in maize and wheat, where alcohol-soluble protein is formed by a single polypeptide chain connected by intramolecular disulfide bonds, while glutelin is formed by multiple polypeptide chains connected by disulfide bonds [51]. Alcohol-soluble protein and glutelin in plant seeds lack essential amino acids for humans, thus their nutritional value is relatively low. However, they find wide applications in other fields [52]. Alcohol-soluble proteins have lipid resistance, heat resistance, and water resistance. For example, wheat storage proteins provide a cohesive network structure to dough, maintaining gas and resulting in soft baked goods, thereby improving the quality of processed foods [53]. Modified enzymatic hydrolysis of foxtail millet alcohol-soluble protein produces foxtail millet alcohol-soluble protein peptides, which have been found to possess anti-inflammatory activity and antioxidant capabilities. Alcohol-soluble proteins from maize can be used as a pharmaceutical sugar coating and applied in drug sustained-release agents [54]. They can also prevent food oxidation and be processed into films with preservation functions [55]. These examples demonstrate the broad application prospects of plant storage proteins and provide new insights for related research on storage proteins in caper seeds.

Based on the analysis of SDS-PAGE electrophoretic profiles, it was observed that the protein fractions from the eight provenances had relatively small molecular weights, and there were differences in protein bands among different provenances. Alcohol-soluble proteins in seeds are not influenced by factors such as growing environment and seed treatment, and they are closely related to the genetic characteristics of the varieties. Therefore, gel electrophoresis analysis of alcohol-soluble proteins in seeds is widely used for variety identification, genetic breeding, and other purposes [56, 57]. Chen et al. [58] analyzed alcohol-soluble proteins in nine varieties of Chinese cabbage seeds and found certain differences in the protein bands among the nine varieties, while some varieties showed similarities. Alcohol-soluble protein analysis revealed genetic differences among Chinese cabbage varieties to some extent. In the results of this experiment, YKL had a specific band in the alcohol-soluble protein profile, while the bands of alcohol-soluble proteins from other provenances were almost identical, indicating relatively small genetic differences among these eight districts of caper seeds. Proteins, as products of gene expression, reflect genetic differences through their profile variations. By combining SDS-PAGE profiles of caper seeds from more provenances, it would be possible to achieve identification of genetic resources, variety authenticity, and seed purity. Furthermore, it is worth noting that although glutelin and alcohol-soluble proteins have relatively high contents in caper seeds, their bands are limited in number. This phenomenon may be attributed to the low solubility of these proteins in the buffer or the partial absence of certain proteins in the gel [59].

Considering that the primary nutritional component in caper seeds is crude fat, the crude fat content serves as the key indicator for screening germplasm resources. Based on the comprehensive PCA analysis results, it is possible to preliminarily identify YKL and YKG as high-quality seed sources. These two provenances were correlated with most of the morphological and nutritional component indicators, and they have the highest crude fat content and seed vitality.

## Conclusions

This study concluded that there were significant differences in the morphological characteristics and major nutritional components of caper seeds among from different provenances. There was a positive correlation between seed morphological characteristics and longitude and altitude. The PCA analysis showed that the caper seeds provenances, which were distributed in geographically distant areas, were clustered, and two promising sources were preliminarily identified. From a genetic perspective, the variation in representative traits among different provenances is the result of the combined effects of environmental influences and genetic variations (e.g., gene flow and geographic isolation). However, based solely on these results, it is not possible to fully differentiate the genetic structure and variation patterns of caper. In order to scientifically define the germplasm resource regions and the geographic variation patterns of caper, further research using molecular marker technology and germplasm experiments are required.

## Materials & methods

The experimental materials were sourced from different regions in Xinjiang, China. The seed populations, along with their corresponding geographical locations, are detailed in Table 5. After collection and removal of impurities, the samples were stored separately in refrigerators at -20 °C and -80 °C. The protein solutions extracted by the Osborne method were stored in a refrigerator at -80 °C.

**Table 5:** The geographical locations of different provenances

Sequence number	Provenance	Latitude(N)	Longitude(E)	Altitude(m)
1	AKS (Aksu)	41.18°	80.27°	1019
2	HYW (WubaoTown,Yizhou District,Hami)	42.88°	92.85°	515
3	HM (Hami)	42.83°	93.52°	734
4	TQQ (Qiquanhu Town,Turpan)	43.15°	89.42°	862
5	TLF (Turpan)	42.96°	89.20°	816
6	TGS (S202,GaochangDistrict, Turpan)	43.13°	89.46°	853
7	YKL (KarayagaqiTownship, Yining County)	44.10°	81.52°	1101
8	YKG (G218,KashiTown,Yining County)	43.68°	82.06°	846

Yingbao Sun formally identified *Capparis spinosa* L. This plant materials have been deposited at the herbarium of the South China Botanical Garden, Chinese Academy of Sciences, with voucher code IBSC 0134349.

### Determination of seed morphological index

Determination of 1000-grain weight: 1000 seeds from different provenances were randomly selected and weighed (accurate to 0.0001 g), and each origin was repeated three times. One seed of each origin was weighed randomly, and a total of 15 sets of data were weighed. The length, width, and thickness of seeds and fruits were measured with the help of Vernier calipers. Seed moisture content was determined by low temperature constant drying method, weigh 3 g of seeds into the drying oven, set the temperature to 105 °C. The seeds were dried for 2 h and weighed. Then the seeds were weighed after 0.5 h of further drying. Until there was no more change in seed weight, the final result was recorded [60].

The particle size can be used to measure the overall size of the seed, and the particle size (D_g_) of the caper seed was calculated by three basic dimensions (seed length: L; width: W; thickness: T):

$${\mathrm{D}}_{\mathrm{g}}={\left(\mathrm{LWT}\right)}^{1/3}$$

Sphericity (ø) can be used to judge the shape of caper seeds, and the calculation formula is:

$$\oslash =\frac{\text{(LWT)}}L^{1/3}$$

Seed surface area (S) and volume (V) can be calculated as follows [61, 62]:

$${\mathrm{S}=\uppi {\mathrm{D}}_{\mathrm{g}}}^{2}$$

$$\mathrm{V}=\frac{\pi {\mathrm{WTL}}^{2}}{6\left(2\mathrm{L}-\sqrt{\mathrm{WT}}\right)}$$

### Determination of seed nutrients

#### Crude fat extraction

Crude fat content was determined by Soxhlet extraction method (GB 2906–82) [63]. Initially, weighed 3 g of caper seeds from different regions, respectively, with three sets of replicates for each region's seeds. The weighed seeds were placed in an oven at 105 °C and dried for 2 h before removal. After drying, the samples were thoroughly ground in a mortar and then transferred into a filter paper cartridge. The filter paper cartridge was subsequently placed into the extraction tube. In a Soxhlet extraction flask containing three glass balls, approximately half the volume of petroleum ether was added. Next, extraction was performed on a water bath (approximately 80 °C) for 8 h. The extraction was considered complete when there was no trace of oil left in the extraction tube, as detected by filter paper. After the extraction, the petroleum ether in the extraction bottle was recovered by distillation on a water bath. The extraction bottle was removed and the residual petroleum ether was evaporated in a boiling water bath. The extraction bottle with crude fat was placed in an oven at 105 °C to dry for 1.5 h, and weighed after cooling, accurate to 0.0001 g. The round-bottom flasked with grease were dried for 0.5 h, cooled to room temperature and then weighed. When the weight remained the same, the results were recorded. The added weight of the extraction bottle was the crude fat weight. The formula for calculating crude fat content is as follows:

$$\mathrm{Crude\, fat }\left(\mathrm{\%}\right)=\frac{\mathrm{Net\, weight\, of\, round\, bottom \,flask}\left(\mathrm{g}\right)+\mathrm{crude\, fat\, weight}-\mathrm{Net\, weight\, of\, round\, bottom\, flask}(\mathrm{g})}{\mathrm{Seed\, dry\, weight}(\mathrm{g})}$$

### Determination of soluble sugar content

Soluble sugar content was determined by phenol-concentrated sulfuric acid method. The standard curve was made and standard solutions were prepared by referring to the literature [64]. 0.1 g of different regions caper seed powders was weighed and they were put into test tubes. 20 mL of distilled water was added and it was put in an ultrasonic extractor for 20 min first. Then the caper seed powders were extracted in a boiling water bath for 30 min. The extract was filtered into a 25 mL volumetric flask, and the volume was adjusted to the mark. 0.5 mL of sample solutions of different origins were transferred into test tubes and 0.5 mL of distilled water was added. After adding 1 mL of 50 g/L phenol solution and 5 mL of concentrated sulfuric acid in sequence, the absorbance was measured at a wavelength of 490 nm. The amount of glucose was obtained from the regression equation, and the content of soluble sugar in the sample was calculated according to the following formula (C: Glucose content found by regression equation; V: Volume of extract; V_1_: Aspirated volume of sample solution; m: Weigh the mass of the sample):

$$\mathrm{Soluble\, sugar\, content }=\frac{\mathrm{C}\times \mathrm{V}}{{\mathrm{V}}_{1 }\times \mathrm{m}\times {10}^{6}}$$

### Determination of soluble protein content

Soluble protein content was determined by Coomassie brilliant blue method, a standard curve was made according to the literature and a regression equation was obtained based on the standard curve [65, 66]. Crushed caper seeds from different places were weighed at 0.1 g and placed in a test tube. Then 10 mL of distilled water was added. After being treated with an ultrasonic extractor for 30 min, the obtained homogenate was placed in a centrifuge at 4000 rpm for 10 min. The obtained supernatant was placed in a 10 mL volumetric flask, and distilled water was added to the volume to the mark. Using a pipette, transfer 0.5 mL of the solution from each sample into a test tube, and add 0.5 mL of distilled water to each tube separately. Subsequently, 5 mL of Coomassie brilliant blue G-250 solution was added, and the absorbance was measured at 595 nm. The protein content was derived from the regression equation, and the protein content was obtained by substituting into the formula.

### Determination of starch content

Starch content was determined by acid hydrolysis method [67]. Acid hydrolysis, also known as acid saccharification, was a method in which acid was used as a catalyst to hydrolyze starch into glucose at high temperature. Different regions' caper seed powders, weighing 0.1 g each, were placed in 10 mL centrifuge tubes. Distilled water (1 mL) was added, followed by the sequential addition of hot ethanol solution (80%) (5 mL). The mixture was shaken thoroughly and left to stand for 5 min. Then centrifuged at 2,500 rpm for 5 min and the supernatant was discarded. It was extracted once more with 6 mL of 80% ethanol solution and the supernatant was poured off again. The residue was added with 1 mL of distilled water and 6 mL of 52% perchloric acid solution and was stirred for 10 min. After being centrifuged at 2,500 rpm for 10 min, the supernatant was transferred to a 10 mL volumetric flask. The volume was adjusted to the mark with distilled water. The soluble sugar content determination method was used as a reference to measure absorbance and calculate the method.

### Protein extraction and determination using Osboren fractionation

**Fig. 7:**
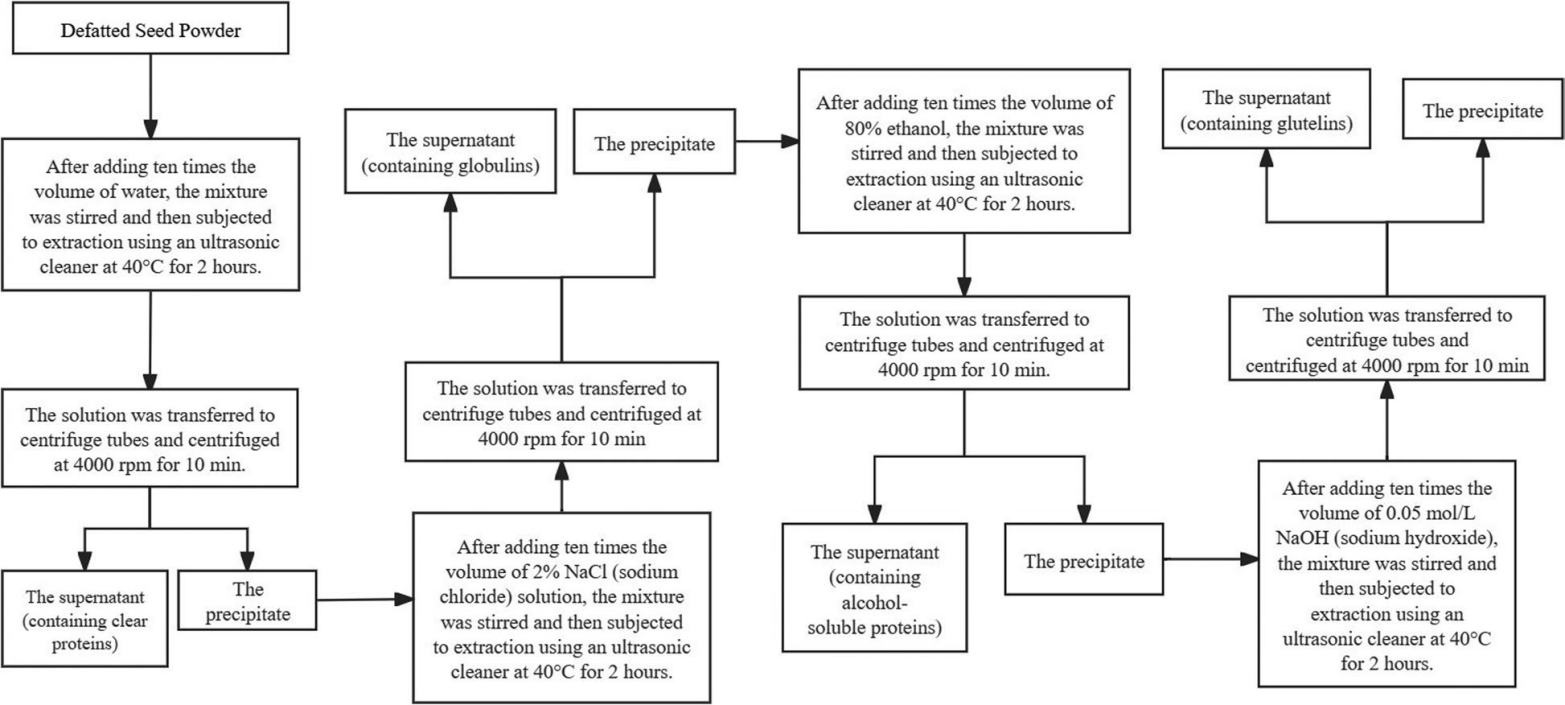
Extraction process of graded protein from caper seeds [68]

The seeds of eight different caper provenances were ground and placed in filter paper cylinders. They were then subjected to extraction using a Soxhlet apparatus for a duration of 6 h to remove the crude fat from the seeds. Following the process outlined in Fig. 7, protein extraction from the seeds of different provenances was performed using Osboren fractionation [67]. The protein content of each fraction was determined using the Coomassie Brilliant Blue method. The extracted protein solution was freeze-dried into a dry powder and stored in a -20 degrees Celsius refrigerator.

### SDS-PAGE analysis of caper seed proteins

SDS-PAGE analysis was performed on the protein fractions obtained from caper seeds using the Osborne fractionation method. A 12% separating gel and a 4% stacking gel were prepared. A protein solution of 2 mg/mL was mixed with a 1:1 volume ratio of 2 × sample buffer, boiled for 3 ~ 5 min, and then centrifuged at 4000 rpm for 10 min. Four microliters of the supernatant were loaded onto the gel for constant current gel electrophoresis. The gel was initially run at a constant voltage of 80 V for 15 min and then at 120 V for 50 min, followed by completion of the electrophoresis. The protein gel was washed with deionized water and then stained overnight with a staining solution on a shaker. Subsequently, the gel was destained for 4 h using a destaining solution (placing a piece of tissue paper next to the protein gel can aid in destaining). Finally, the gel was photographed using the SmartGel 5000 imaging system with appropriate camera settings.

### Statistical analysis

The raw data was processed using EXCEL 2019 to establish the original data document. The statistical software SPSS Statistics 26 was employed to conduct correlation analysis on the data.

The correlation heatmaps and PCA analysis biplot were generated using Origin 2021 software.

### Supplementary Information

**Additional file 1: Table S1.1 **is the first supplementary document to Table 1 in the main text. **Table S1.2.** is the second supplementary document to Table 1 in the main text. **Table S2.** is the supplementary document to Table 2 in the main text.

## Data Availability

All data generated or analysed during this study are included in this article and its supplementary information files.
